# Supplementary material for: IL-6 and TNF-α salivary levels according to the periodontal status in Portuguese pregnant women
Source: PeerJ. 2018 May 4;6:e4710. doi: 10.7717/peerj.4710 (PMC5937472; doi:10.7717/peerj.4710)
Supplement: Supplemental Information 1 [file peerj-06-4710-s001.docx]

**IL-6 and TNF-α salivary levels according to the periodontal status in Portuguese pregnant women**

Number _________

**Socio-demographic characteristics**

Age _______

Education level

- 1. Basic/Middle
  2. Higher

Marital Status

1. Married
2. Single

Occupation

| 1. Employed | 1. Unemployed |
| --- | --- |

**Oral Behaviours**

Toothbrush frequency

| 1. One time daily | 2. two or more times daily |  |
| --- | --- | --- |

Dental floss usage

| 1. Yes | 1. No |
| --- | --- |
